# Oral Zinc-Rich Oyster Supplementation Corrects Anemia in Rats

**DOI:** 10.3390/nu15214675

**Published:** 2023-11-04

**Authors:** Yen-Hua Chen, Hui-Lin Feng, Yu-Cheng Lu, Sen-Shyong Jeng

**Affiliations:** 1 Institute of Food Safety and Risk Management, College of Life Sciences, National Taiwan Ocean University, Keelung 20224, Taiwan; yhchen0912@mail.ntou.edu.tw; 2 Department of Food Science, College of Life Sciences, National Taiwan Ocean University, Keelung 20224, Taiwan; fhl1430@gmail.com (H.-L.F.); awdrg134679@gmail.com (Y.-C.L.)

**Keywords:** zinc supplementation, anemia, red blood cells, oysters, hard clam, phenylhydrazine-(PHZ) induced rats, 5/6-nephrectomized rats, chronic kidney disease (CKD)

## Abstract

This study investigates the impact of various zinc supplementation methods on anemia in rats induced by phenylhydrazine (PHZ) and in 5/6-nephrectomized anemic rats. We compare oral zinc sulfate (ZnSO_4_) supplementation, oyster *Crassostrea gigas* supplementation, and hard clam *Meretrix lusoria* supplementation on red blood cell (RBC) levels. Oral zinc-rich oyster supplementation (2.70 mg Zn (30 g oyster)/day/rat) effectively corrects anemia in both experimental groups. Rats orally fed oysters for four days exhibit similar effectiveness as those receiving a single ZnSO_4_ injection (0.95 mg Zn (4.18 mg ZnSO_4_⋅7H_2_O)/rat). In contrast, oral ZnSO_4_ supplementation (2.70 mg Zn (11.88 mg ZnSO_4_⋅7H_2_O)/day/rat) does not significantly increase RBC levels, suggesting better zinc absorption from oysters. A placebo group of anemic rats supplemented with hard clams, similar in composition to oysters but much lower in zinc, did not change RBC counts. This supports oysters’ high zinc content as the key to correcting anemia. Oysters also contain high iron levels, offering a potential solution for iron-deficiency anemia while supporting bone marrow erythropoiesis. In summary, oral oyster supplementation emerges as an effective strategy to correct anemia in rats with added zinc and iron support for erythropoiesis.

## 1. Introduction

Anemia represents the most prevalent blood disorder [1], arising from two primary factors: insufficient nutrition (including deficiencies in iron, vitamin B12, folate, and others) and compromised health conditions (such as chronic kidney disease (CKD) or bone marrow disorders) [2,3,4]. Iron deficiency remains the leading cause of anemia across various age groups, whereas CKD takes precedence as the primary cause of anemia among the elderly [5,6,7]. The treatment approach for nutritional anemia primarily involves supplying deficient nutrients, while CKD patients often necessitate intervention through recombinant human erythropoietin (rhuEPO) [8,9,10].

Zinc, a crucial trace element, plays a multifaceted role in vital biological functions. Zinc deficiency affects systemic growth, metabolism, development of connective tissue, bone and teeth, immune responses, cytokine production, and endocrine regulation [11,12,13,14,15]. However, zinc deficiency alone does not result in anemia and it may need to cooperate with other factors to lead to anemia. In most cases, zinc deficiency coexists with iron deficiency, and to a lesser extent, zinc deficiency may cooperate with other factors to lead to anemia [7]. In many pregnant women and infants suffering from anemia, several reports indicate that zinc supplementation combined with iron therapy can increase hemoglobin levels and improve iron indexes more than iron alone [16,17,18,19,20,21,22,23]. Beyond correcting zinc deficiency, zinc supplementation may also influence anemia by inducing erythropoiesis through a process known as zinc-induced erythropoiesis [24]. Research has shown that zinc has the potential to stimulate erythropoiesis in various animal models, including fish and rats [24,25]. This ‘zinc-induced erythropoiesis’ appears to be a shared mechanism across diverse animal species [25,26]. 

Phenylhydrazine hydrochloride (PHZ) has been shown to induce oxidative denaturation or hemolysis of red blood cells. Rats treated with PHZ have proven to be a valuable experimental model for investigating anemia and assessing the hematological responses to novel agents [27,28,29]. In a previous study, the impact of zinc supplementation on PHZ-induced anemia in rats was investigated. It was observed that administering a specific quantity of ZnSO_4_ solution, exceeding 2.8 mg Zn/kg body weight, resulted in a significant increase in their RBC levels. Remarkably, within just two days, these injections elevated the RBC levels in the rats from 60% to 88% of those observed in normal rats [25]. Additionally, 5/6-nephrectomized rats, which serve as an experimental model for CKD by mimicking the progression of renal failure following the loss of renal mass, closely resembling the situation in humans, are commonly employed in research [30,31,32]. Our investigations revealed that administering the same zinc dosage (2.8 mg Zn/kg body weight) to 5/6-nephrectomized uremic anemic rats led to an increase in RBC levels from 85% of the control to 95% within a single day, sustaining this improvement for four days [24]. These findings strongly suggest that zinc supplementation holds significant promise as a potential approach for correcting anemia in these experimental models.

In the aforementioned studies [24,25,26], zinc supplementation to rats was primarily administered through injections to ensure optimal zinc absorption. However, for practical applications, oral supplementation offers greater convenience [33,34]. Zinc sulfate has traditionally been the most common and cost-effective zinc compound used, typically in injection form. Nevertheless, studies have indicated that organic zinc compounds are more readily absorbed when taken orally as compared to inorganic zinc compounds [35,36].

Foods with high protein content are also rich in zinc content, whereas those foods containing mostly carbohydrates are much lower in zinc content [15,37]. Among natural food sources, oysters emerge as an exceptional zinc source, containing 9.0 mg of zinc per 100 g in Pacific oysters and reaching 74.7 mg of zinc per 100 g in Atlantic oysters in North America [38]. In Taiwan, the average zinc content in oysters was found to be 12 mg per 100 g [39]. While the zinc content in oysters can vary significantly depending on location and season, they consistently have the highest zinc levels among major dietary foods. In comparison, raw beef, pork, and chicken contain 4.2, 2.7, and 0.7 mg of zinc per 100 g, respectively [38]. Freshwater fish, marine fish, and various invertebrates (including shrimp, crab, clam, and squid) contain 0.5–2.4, 0.2–0.6, and 0.6–1.4 mg of zinc per 100 g, respectively [40]. Oysters, therefore, stand out as a natural food source with the highest zinc content. In a study evaluating the biological availability of zinc in food items from both plant and animal sources, O’Dell et al. [41] noted that when chickens were used as test animals, oysters demonstrated the highest biological availability of zinc.

Given the high zinc content and remarkable biological availability of zinc in oysters, our current study was designed to investigate their effects on anemic rats. We conducted experiments involving two groups of anemic rats: one induced with PHZ and the other 5/6-nephrectomized to induce anemia. We administered oral doses of oysters and compared their effects with those of ZnSO_4_, analyzing their impact on RBC levels and other hematologic indices in the rats. Our findings conclusively demonstrate that oral oyster supplementation effectively corrects anemia in these rats.

## 2. Materials and Methods

### 2.1. Animal Acquisition and Housing

We obtained both normal and 5/6-nephrectomized adult male Sprague–Dawley rats at 8 weeks of age from BioLASCO Taiwan Co., Ltd., Taipei, Taiwan. This company is the authorized distributor for research models from Charles River Laboratories in Taiwan and Southeast Asia. The 5/6 nephrectomy procedure, following Surgery Code: 56NEPHRES, was conducted using a two-stage standard method in accordance with the protocols established by Charles River North American Research Models in Wilmington, MA, USA.

The rats were housed in our in-house animal facility, with environmental conditions maintained at 22 ± 3°C and 55 ± 5% relative humidity, following a 12-h light–dark cycle. They had unrestricted access to tap water and the MFG diet sourced from Oriental Yeast Co. Ltd. (Nagano, Japan), which was purchased through BioLASCO Taiwan Co., Ltd. We conducted an analysis of the dietary zinc content and found it to be 45 mg Zn/kg of the diet. All animal procedures were reviewed and approved by the Institutional Animal Care and Use Committee at National Taiwan Ocean University (Approval No. 111019, granted on 10 December 2022).

### 2.2. Zinc Supplementation and Dietary Preparation

The rats were administered ZnSO_4_ solution via intraperitoneal injection at a dose of 2.8 mg Zn/kg body weight [25], equivalent to 0.95 mg Zn per rat, considering a mean body weight of 339 ± 17 g (mean ± SD), or 4.18 mg of ZnSO_4_⋅7H_2_O per rat.

Oral ZnSO_4_ supplementation was achieved by applying 0.5 mL of a 1.8 mg Zn (7.92 mg ZnSO_4_⋅7H_2_O)/mL solution onto a 3-g pellet of the MFG diet. This prepared, compact, and moistened pellet provided an additional 0.9 mg of zinc supplementation. Three such pellets were offered to each rat, resulting in a daily zinc supplementation of 2.70 mg/rat.

Oysters *Crassostrea gigas* and hard clams *Meretrix lusoria* were procured from Chiayi County through the local Keelung City market. The zinc content in oysters was determined to be 91 μg/g in fresh tissue, while clams contained 14 μg/g in fresh tissue. Approximately 900 g of shucked oyster or clam meat was collected, comprising around 180 or 360 individuals. Oysters weighed approximately 5–6 g each, whereas hard clams weighed 2–3 g each. The oyster and clam meat were finely minced and stored at −20°C. Subsequently, the frozen samples underwent freeze-drying using a lyophilizer, resulting in dried samples weighing 151 g. For the preparation of experimental pellets, the dried sample was mixed with 3.5% cellulose (5.31 g) and 15% water. Pellets, weighing approximately 1.6 g each, were formed using a compressor. The oyster pellet contained 563 μg/g of zinc, while the clam pellet contained 88 μg/g of zinc.

To supply a total of 2.70 mg of zinc to a rat, three oyster pellets, each weighing 1.6 g with a zinc content of 563 μg/g (or 30 g of oysters with a zinc content of 91 μg/g), were administered. Normally, a rat consumes 30 g of the MFG diet per day. To ensure that the rats consumed all the oyster or clam meat, it was administered prior to offering the MFG diet. Once the rats had completed their consumption of the oyster or clam pellets, the MFG diet was subsequently provided.

### 2.3. Effects of Different Zinc Supplementation Methods on RBC Levels of PHZ-Induced Anemic Rats

After 1 week of acclimatization in our laboratory, normal rats (body weight, 339 ± 17 g, mean ± SD) (*n* = 30) received either sterilized normal saline (0.9% NaCl, *n* = 6) or a single dose of PHZ hydrochloride (Sigma Chemical Co., St. Louis, MO, USA) in sterilized normal saline (*n* = 24) through intraperitoneal injection at 60 mg/kg body weight. After 2 days, the PHZ-induced anemic rats (day 2) were divided into four groups (*n* = 6 each) and supplemented with zinc in different ways: (a) without supplementation (control, saline injection); (b) injection of ZnSO_4_ (0.95 mg Zn/rat, equivalent to 4.18 mg ZnSO_4_⋅7H_2_O/rat); (c) fed with ZnSO_4_ (2.70 mg Zn/day/rat, equivalent to 11.88 mg ZnSO_4_⋅7H_2_O/rat); and (d) fed with oyster (2.70 mg Zn/day/rat, equivalent to 30 g oyster/day/rat). Four days later (day 6) after supplementation, blood was drawn from the rats, and RBC levels and other hematological indices were measured. Three independent experiments were conducted.

### 2.4. Effects of Oral ZnSO_4_ and Oyster Supplementation on RBC Levels in 5/6-Nephrectomized Anemic Rats

In this study, we utilized a cohort of 36 rats, comprising 30 rats subjected to 5/6 nephrectomy, while 6 rats remained untreated. After a 25-day period of acclimatization in our laboratory, the normal rats (Group A) exhibited an average body weight of 344 ± 10 g (mean ± SD), while the 5/6-nephrectomized rats (*n* = 30) developed uremic anemia with a body weight of 320 ± 11 g (mean ± SD). The 5/6-nephrectomized rats were subsequently divided into five subgroups: B-a: No supplementation; B-b: Supplemented with ZnSO_4_ (1.35 mg Zn/day/rat, equivalent to 5.94 mg ZnSO_4_⋅7H_2_O/rat); B-c: Supplemented with ZnSO_4_ (2.70 mg Zn/day/rat, equivalent to 11.88 mg ZnSO_4_⋅7H_2_O/rat); B-d: Supplemented with oyster (1.35 mg Zn (equivalent to 15 g oyster/day/rat); B-e: Supplemented with oyster (2.70 mg Zn/day/rat, equivalent to 30 g oyster/day/rat). At 2, 4, 6, or 8 weeks after supplementation, blood samples were collected from the rats to measure RBC levels and other hematological parameters. Two separate experiments were conducted.

### 2.5. Comparison of the Effect of ZnSO_4_, Oyster, or Clam Supplementation on RBC Levels of 5/6-Nephrectomized Rats

Among 24 normal rats, 18 rats underwent surgical removal of 5/6 of their kidneys, while 6 rats received no treatment (group A). After 25 days, the 5/6-nephrectomized rats (*n* = 18) were further divided into three subgroups: (B-a) fed with ZnSO_4_ (2.70 mg Zn/day/rat, equivalent to 11.88 mg ZnSO_4_⋅7H_2_O/rat); (B-b) fed with oyster (2.70 mg Zn/day/rat, equivalent to 30 g oyster/day/rat); (B-c) fed with hard clam (0.42 mg Zn/day/rat, equivalent to 30 g hard clam/day/rat). At 0, 2, 4, 6, or 8 weeks after supplementation, blood was drawn from the rats, and RBC levels and other hematological indices were measured. 

### 2.6. Hematological Analysis

Hematological parameters were evaluated using an automated hematological assay analyzer (Cell Excell 500; Danam Electronics, Dallas, TX, USA).

### 2.7. Determination of Zinc Concentration

After collecting whole blood from the rats, 0.5 mL of the sample was centrifuged at 2000× *g* for 10 min, and the resulting supernatant was directly employed for measuring the plasma zinc concentration. In the case of determining zinc levels in bone samples (specifically the femur, prepared through dry ashing), the bone ash was homogenized using a 50 mM Tris-buffer solution (pH 8.0) with 6 mM EDTA. Subsequently, the homogenate was centrifuged at 10,000× *g* for 30 min, and the resultant supernatant was utilized for zinc determination [12]. The quantification of zinc levels was accomplished using an atomic absorption spectrophotometer (Z-8100, Hitachi, Tokyo, Japan) by the method of Fuwa et al. [42].

### 2.8. Statistical Analysis

The data are presented as the mean ± standard deviation (SD). To assess the statistical significance of the experimental results, we performed a one-way analysis of variance (ANOVA) followed by the least significant difference post hoc test, utilizing SPSS 22.0 (SPSS Inc., Chicago, IL, USA).

## 3. Results

### 3.1. Effects of Various Zinc Supplementation Methods on RBC Levels in PHZ-Induced Anemic Rat

**Figure 1:**
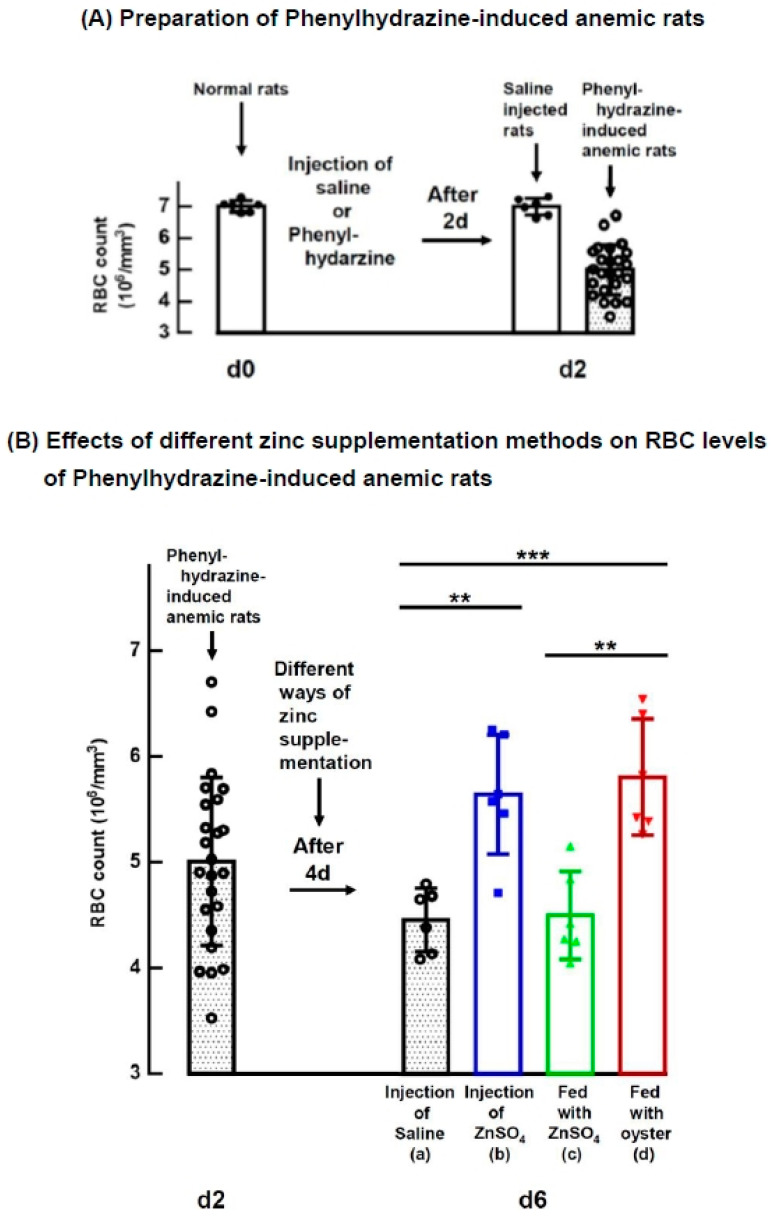
Effects of Different Zinc Supplementation Methods on RBC Levels of PHZ-Induced Anemic Rats. (**A**) Normal rats (*n* = 30) were injected with either saline (*n* = 6) or PHZ (*n* = 24). After 2 days, the PHZ-injected rats developed anemia. (**B**) The PHZ-induced anemic rats (d2) were divided into four groups (*n* = 6 for each group) and supplemented with zinc in different ways: (a) without supplementation (control, injection of saline); (b) injection of ZnSO_4_; (c) fed with ZnSO_4_; and (d) fed with oyster. The amount of zinc supplemented in each group is shown in Table 1. Four days (d6) after supplementation, rat blood was withdrawn, and RBC levels and other hematological indices of the rats were measured (** *p* < 0.01, *** *p* < 0.001). This figure represents three independent experiments.

Normal rats were induced into anemia by administering PHZ, as illustrated in Figure 1A. Subsequently, the effect of four different zinc supplementation methods on the RBC levels of these anemic rats was investigated, and the results are presented in Figure 1B. Further details regarding the hematology of the normal rats, PHZ-induced rats, and anemic rats with different zinc supplementations are provided in Table S1.

Figure 1A demonstrates that a single bolus injection of PHZ (60 mg/kg body weight) in normal rats led to a significant decrease in RBCs, reducing to 72 ± 11% of the normal rat levels (*p* < 0.001) within two days. Figure 1B displays the RBC levels four days after zinc supplementation. Among the rats injected with saline (group a), RBC levels dropped further to 64 ± 4% of the normal rat levels. However, the rats injected with ZnSO_4_ (group b) and those fed with oysters (group d) exhibited significantly higher RBC levels compared to the saline-injected rats (group a), reaching 81 ± 8 and 83 ± 8% of the normal rat levels, respectively (*p* = 0.002 and <0.001). Notably, the RBC proportion in ZnSO_4_-fed rats (group c) was significantly lower than that in oyster-fed rats (group d) (65 ± 6 vs. 83 ± 8% of the normal rat levels, *p* = 0.001), even though both groups received the same amount of supplemented zinc, 2.70 mg Zn/day/rat (Table 1).

**Figure 2:**
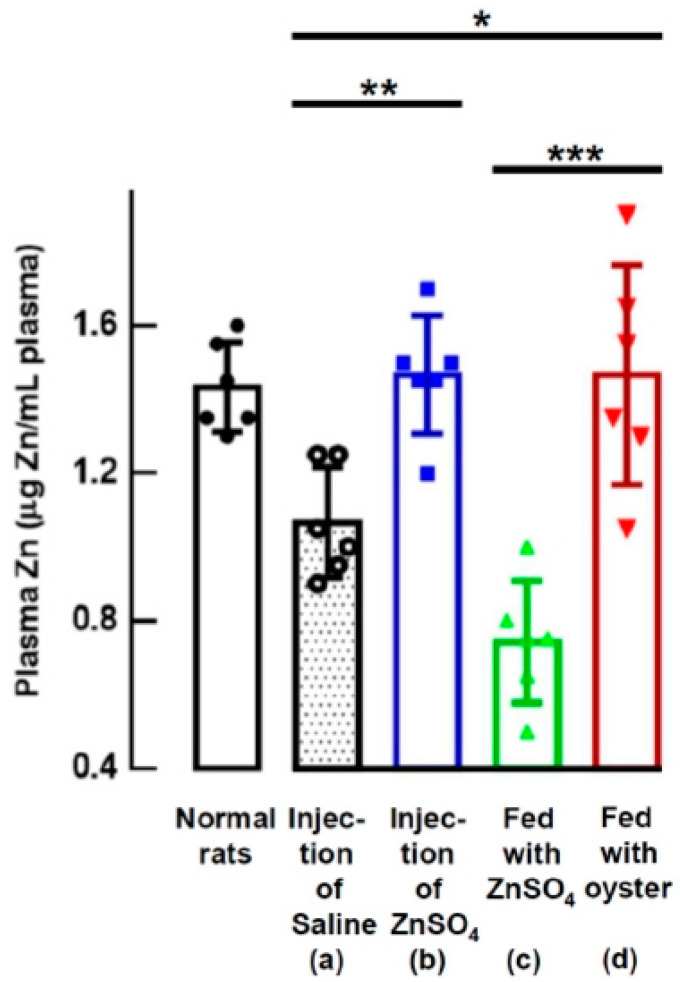
Plasma Zinc Levels Four Days After Zinc Supplementation in PHZ-Induced Anemic Rats. Four days after zinc supplementation in PHZ-induced anemic rats (at d6), the plasma zinc levels of the normal rats and the treated rats were measured (* *p* < 0.05, ** *p* < 0.01, *** *p* < 0.001). This figure represents three independent experiments.

Figure 2 displays the plasma zinc levels four days after zinc supplementation. The results are consistent with the trends observed in the RBC counts. The rats injected with ZnSO_4_ (group b) and those fed with oysters (group d) had significantly higher plasma zinc levels compared to the control rats injected with saline (group a) (1.47 ± 0.16 and 1.47 ± 0.30 vs. 1.07 ± 0.15 µg Zn/mL plasma, *p* = 0.0012, and 0.02053). Furthermore, the plasma zinc levels in ZnSO_4_-fed rats (group c) were significantly lower than in rats fed with oysters (group d) (1.47 ± 0.30 vs. 0.74 ± 0.17 µg Zn/mL plasma, *p* = 0.00087).

### 3.2. Effects of Oral ZnSO_4_ and Oyster Supplementation on RBC Levels in 5/6-Nephrectomized Anemic Rats

**Figure 3:**
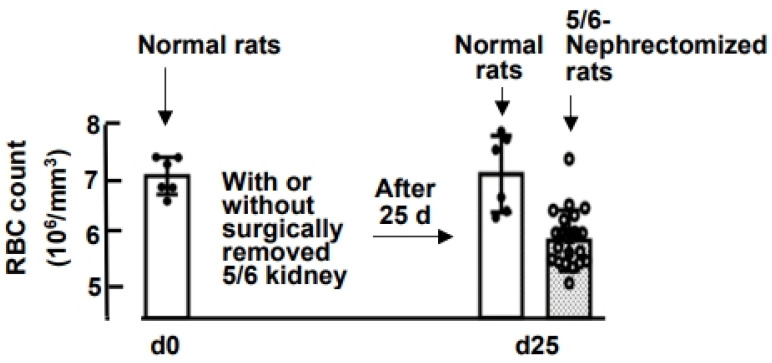
Preparation of 5/6-Nephrectomized Anemic Rats. Among 36 normal rats, 30 underwent. surgical removal of 5/6 of their kidneys. After 25 days, the 5/6-nephrectomized rats became anemic.

**Figure 4:**
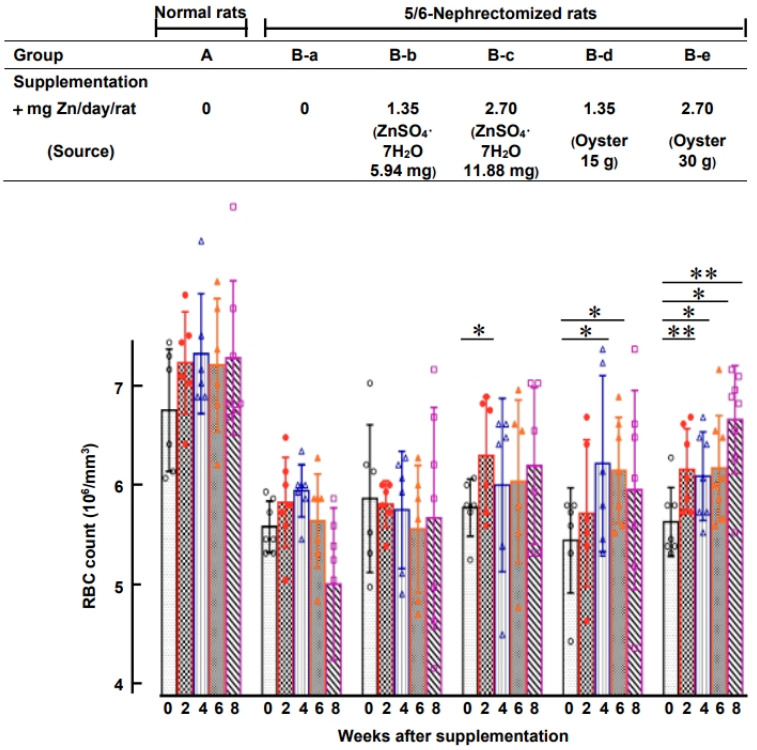
Effect of Oral ZnSO_4_ and Oyster Supplementation on RBC levels of 5/6-Nephrectomized Anemic Rats. The 5/6-nephrectomized anemic rats (*n* = 30) from Figure 3 were divided into five groups (*n* = 6 for each group) with or without oral supplementation of ZnSO_4_ or oyster, as shown in the figure. For comparison, normal rats from the same batch (*n* = 6) were fed a standard diet without zinc supplementation throughout the experiment. At 0, 2, 4, 6, or 8 weeks after supplementation, rat blood was drawn, and RBC levels and other hematological indices were measured (* *p* < 0.05, ** *p* < 0.01 compared with the 0-week group). This figure represents two independent experiments.

Figure 3 illustrates that 25 days after 5/6 nephrectomy in normal rats, the RBC levels of the 5/6-nephrectomized rats decreased significantly to only 84 ± 6% of the pre-surgery levels ((5.75 ± 0.40) × 10^6^ vs. (6.81 ± 0.56) × 10^6^ cells/mm^3^). Subsequently, Figure 4 presents the effect of oral ZnSO_4_ and oyster supplementation on RBC counts in rats with established uremic anemia, and the hematology data for these rats are summarized in Table S2. The results indicate that oral ZnSO_4_ supplementation had limited impact on the recovery of RBC counts, regardless of whether the supplemented zinc dose was 1.35 mg Zn/day/rat or 2.70 mg Zn/day/rat (groups B-b and B-c). However, in the case of administering 2.70 mg Zn (30 g oysters)/day/rat to the 5/6-nephrectomized rats (group B-e), RBC counts after 2, 4, 6, and 8 weeks of supplementation were significantly higher than those in the 0-week group (91 ± 5, 91 ± 6, 91 ± 8, and 97 ± 7% of the normal rat levels, respectively, *p* = 0.003, 0.019, 0.019, and 0.001). This finding suggests that an adequate amount of oral oyster supplementation, exceeding 2.70 mg Zn (30 g oyster)/day/rat, effectively corrects anemia. In contrast, when the oyster supplementation was halved (1.35 mg Zn (15 g oyster)/day/rat, group B-d), the improvements in RBC counts were not as pronounced. 

**Figure 5:**
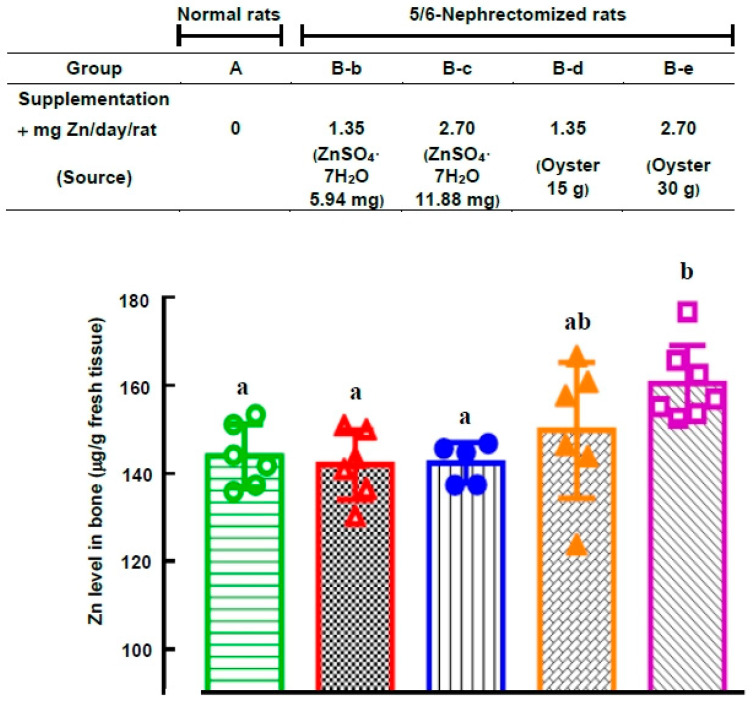
Comparison of Zinc Levels in the Bones of Normal Rats and 5/6-Nephrectomized Rats Supplemented with Oral ZnSO_4_ or Oyster at the End of the Experiment. The normal and 5/6-nephrectomized rats from Figure 4 were sacrificed at d 81 (8 weeks after supplementation), and the zinc levels in their bones were measured. Values with different letter superscripts are significantly different at *p* < 0.05.

According to Chen et al. [26], excess zinc fed to 5/6-nephrectomized rats led to zinc storage in bones without significant changes in plasma zinc levels. In our experiment, we also found no significant difference in plasma zinc levels between different zinc supplementation methods (Table S3). However, Figure 5 demonstrates that after 8 weeks of supplementation, the group (B-e) (oral oyster supplementation of 2.70 mg Zn (30 g oyster)/day/rat) exhibited significantly higher zinc levels in bones (160.5 ± 8 µg Zn/g fresh tissue) compared to all the other groups (142–150 µg Zn/g fresh tissue). These results indicate that only in the B-e group, there was an excess of zinc stored in bones. Moreover, when the same amounts of zinc (2.70 mg Zn/day/rat) were orally administered through ZnSO_4_ (group B-c), the rats absorbed more zinc from oral oysters than from oral ZnSO_4_.

### 3.3. Comparison of Oyster and Hard Clam Supplementation Effects on RBC Levels in 5/6-Nephrectomized Rats

In the above experiment (Figure 4), it becomes evident that an appropriate amount of oyster supplementation can effectively correct uremic anemia in rats. However, the precise reason for this effect remains uncertain, as it is not clear whether it solely stems from the high zinc content in oysters or if other components within oysters also contribute to stimulating erythropoiesis. Both hard clams and oysters belong to the bivalve family and share a similar chemical composition [43], except that hard clams contain a very minimal amount of zinc [39]. To develop further into this matter, we selected hard clams as a placebo group to explore whether they could also correct anemia in rats.

**Figure 6:**
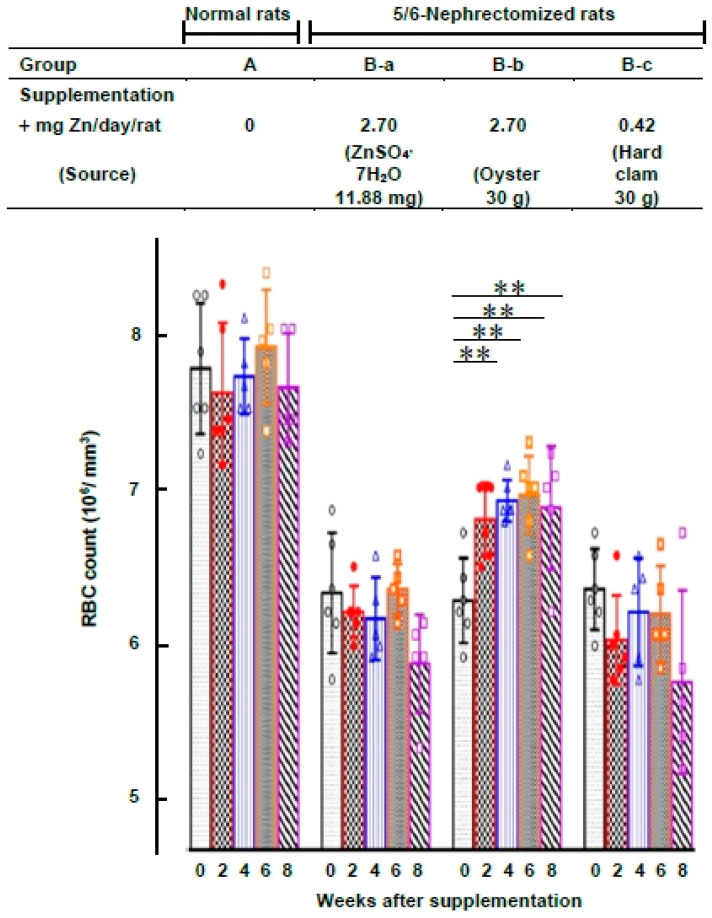
Comparison of the Effect of ZnSO_4_, Oyster, or Clam Supplementation on RBC Levels of 5/6-Nephrectomized Rats. Among 24 normal rats, 18 rats underwent surgical removal of 5/6 of their kidneys, while 6 rats received no treatment (group A). After 25 days, the 5/6-nephrectomized rats (*n* = 18) were further divided into three subgroups (*n* = 6 for each group) with oral ZnSO_4_, oyster, or hard clam supplementation, as shown in the figure. The difference between group (B-b) and (B-c) is the replacement of oyster with the same amount of hard clam. For comparison, normal rats were fed a standard diet without zinc supplementation throughout the experiment. At 0, 2, 4, 6, or 8 weeks after supplementation, rat blood was drawn, and RBC levels and other hematological indices were measured (** *p* < 0.01 compared with the 0-week group).

We prepared 5/6-nephrectomized rats, and in Figure 6, we compared the effects of oral ZnSO_4_, oyster supplementation, and hard clam supplementation on RBC levels in these rats. The hematology data for these rats are summarized in Table S4. In Figure 6, both groups B-b and B-c received the same quantities of oysters and hard clams (30 g/day/rat), respectively, but with significantly different zinc contents of 2.70 mg Zn/day/rat and 0.42 mg Zn/day/rat, respectively. Our findings indicate that after 2, 4, 6, and 8 weeks, only the oyster-supplemented group (group B-b) exhibited significantly higher RBC counts compared to the baseline group (0-week). In contrast, the groups supplemented with ZnSO_4_ and hard clams (groups B-a and B-c) showed no significant effects in correcting their RBC counts. These results suggest that the positive impact on RBC levels in 5/6-nephrectomized rats is likely due to the high zinc content in oysters.

## 4. Discussion

### 4.1. Reversing Anemia in Rats through Oral Oyster Supplementation

Within this study, we delved into the potential of oral oyster supplementation to counteract anemia in rats. We employed two distinct methods to induce anemia: firstly, short-term anemia was provoked by administering PHZ, manifesting anemia just two days post-injection. Secondly, we emulated the conditions seen in CKD patients by surgically removing 5/6 of the rat’s kidney, resulting in prolonged anemia due to impaired EPO production. This kidney-related dysfunction subsequently led to uremic anemia in the rats after a 25-day period.

In both experiments, oral oyster supplementation demonstrated a remarkable capacity to rectify anemia in the rats. For rats subjected to PHZ-induced anemia, providing 30 g oysters/day/rat (equivalent to 2.70 mg Zn/day/rat) resulted in a substantial increase in their RBC levels. Within four days, their RBC levels rose from 64 ± 4% to 83 ± 8% of the levels observed in normal rats (Figure 1). Likewise, among rats experiencing anemia resulting from 5/6 kidney removal, the administration of the same zinc-rich oysters (30 g oysters/day/rat or 2.70 mg Zn/day/rat) led to a notable upsurge in RBC count, raising it from 84 ± 6% to approximately 91–97% of the levels seen in normal rats. This positive impact manifested within 2 to 8 weeks of consistent supplementation (Figure 4 B-e group and Figure 6 B-b group). These findings strongly suggest that an optimal quantity of oral oyster supplementation, surpassing the threshold of 2.70 mg of Zn (equivalent to 30 g of oysters)/day/rat, can effectively rectify anemia in rats.

### 4.2. Major Factor in Oysters Responsible for Correcting Anemia in Rats

In the experiments of rats subjected to anemia induction through PHZ administration, the group receiving oyster supplementation (Figure 2 group d) exhibited a remarkable increase in plasma zinc levels compared to the control rats that were administered saline injections (Figure 2 group a). This disparity strongly implies a significant accumulation of zinc within the tissues of the oyster-fed group, setting it apart from the control group.

In Figure 5, it is shown that following a consistent 8-week supplementation regimen, rats with 5/6 nephrectomies and diets enriched with oysters showcased notably elevated zinc levels within their bones, outpacing all other groups. This observation underscores the surplus of zinc stored in the bones of anemic rats subsequent to nourishment with zinc-rich oysters. 

To ascertain whether elevated zinc is the pivotal factor driving the capacity of oyster supplementation to rectify anemia in rats, a hard clam group was chosen as a placebo. In Taiwan, the oysters are cultivated in lower coastal areas, and the hard clam is also cultivated in the same sandy flat. Both are bivalves, sharing nearly identical environmental conditions. The average proximate composition of Taiwan’s oysters is as follows: moisture 84.98%, protein 8.61%, lipid 1.02%, glycogen 0.71%, and ash 1.92%. Taiwan’s hard clams exhibit somewhat similar concentrations: moisture 81.31%, protein 11.01%, lipid 0.83%, glycogen 0.64%, and ash 3.08% [43]. The mean concentrations of Zn, Cu and Fe in Taiwan’s oysters were found to be 120.6, 20.4 and 86.1 μg/g fresh tissue, respectively, while in hard clams, these concentrations were 14.9, 5.7 and 136.1 μg/g fresh tissue, respectively. The most significant difference in trace element concentration between oysters and hard clams was observed in zinc. Oysters contained approximately 8 times the Zn content and 4 times the Cu content of hard clams, whereas the Fe content did not exhibit such significant variation [39]. In this study, both oysters and hard clams were provided to 5/6-nephrectomized anemic rats at equal quantities of 30 g per day per rat. However, a substantial disparity in zinc content between the two was evident, with oysters supplying 2.70 mg of zinc per day per rat, while hard clams offered only 0.42 mg of zinc per day per rat (as shown in Figure 6). This marked a 6.4-fold difference in zinc content between the two sources. The 6.4-fold difference in zinc levels observed between oysters and hard clams in our study (91:14 μg/g fresh tissue) is smaller than the 8-fold difference (121:15 μg/g fresh tissue) reported by Hsu et al. [39]. This variation can be attributed to seasonal fluctuations in zinc levels in Taiwan’s oysters.

Given the comparable proximate composition of oysters and clams, alongside minimal differences in Fe concentration, it is highly plausible that the significant zinc difference is the key factor enabling oysters, but not hard clams, to correct anemia. Nonetheless, it is important to acknowledge that beyond high zinc content, the potential presence of other beneficial constituents or synergistic interactions in oysters may also contribute to their erythropoietic effects. Some studies suggest that oysters may enhance the immune system and serve as a rich source of bioactive compounds with various biological activities [44]. Further investigations are warranted to uncover the precise mechanisms underpinning the observed enhancement in RBC levels through oyster supplementation.

### 4.3. The Effectiveness of Zinc Supplementation Is Influenced by Several Key Factors, including the Method of Administration, the Dosage of Zinc, and the Specific Form of Zinc Utilized

As illustrated in Figure 1, a single injection of a ZnSO_4_ solution significantly increased the RBC counts in rats with PHZ-induced anemia, raising them from 72 ± 11% to 81 ± 8% of the levels observed in normal rats within a 4-day period. Similarly, when rats received oral supplementation with oysters, providing 2.70 mg Zn/day/rat, a comparable effect was achieved. This raised the RBC counts of anemic rats from 72 ± 11% to 83 ± 8% of normal levels within the same 4-day period. But the oral supplementation required a larger quantity of zinc compared to the injection dosage, as indicated in Table 1.

In this study, the appropriate oral zinc quantity administered through oysters was determined to be 2.70 mg Zn/day/rat (equivalent to 30 g oysters/day/rat). Figure 4 demonstrates that halving the quantity of oyster supplementation (15 g oysters/day/rat) resulted in less noticeable improvement in rat anemia.

Beyond dosage, the form of zinc also played a crucial role. Figure 4 and Figure 6 indicate that administering the same quantity of zinc through ZnSO_4_ orally (2.70 mg Zn from 11.88 mg of ZnSO_4_/day/rat) did not have any impact on correcting anemia, unlike the effects observed with oyster-derived zinc (2.70 mg Zn from 30 g oysters/day/rat). Clearly, the form of zinc made a significant difference. This distinction is further emphasized by the results in Figure 2, where the group of rats fed oysters (group d) exhibited significantly higher plasma zinc levels compared to those fed ZnSO_4_ (group c) (1.47 ± 0.30 vs. 0.74 ± 0.17 mg Zn/mL plasma, *p* < 0.001). Additionally, when equal quantities of zinc (2.70 mg Zn/day/rat) from either ZnSO_4_ or oysters were orally administered to 5/6-nephrectomized anemic rats (Figure 5, groups B-c and B-e), rats supplemented with oysters displayed higher zinc content in their bones after 8 weeks compared to those supplemented with ZnSO_4_ (160.5 ± 8 vs. 142.4 ± 4.2 μg Zn/g fresh bone, *p* < 0.05). Clearly, zinc from oysters was more readily absorbed than ZnSO_4_.

In the pursuit of correcting anemia through zinc supplementation, oral administration emerges as a practical and safe option. However, the choice of zinc quantity and form is pivotal. 

### 4.4. Oysters and Their Potential Role in Managing Anemia

Dating back to 1932, Coulson et al. proposed that oysters could play a pivotal role in the treatment and prevention of nutritional anemia due to their rich iron and copper content. After analyzing 120 different foods, their research revealed that oysters emerged as an outstanding source of iron and copper in a typical serving. Furthermore, an average serving of oysters, approximately 110 grams, can supply approximately 41% of the recommended daily dietary iron intake, which is roughly 15 mg [45].

As mentioned in the introduction, anemia can be attributed to two primary causes: iron deficiency and impaired kidney function leading to insufficient production of EPO. Oysters not only boast significantly higher levels of zinc but also a substantial iron content. Therefore, oysters offer a unique dietary option that addresses both leading causes of anemia. They provide a solution for iron-deficiency anemia by furnishing ample iron while simultaneously delivering zinc to support bone marrow erythropoiesis in individuals with anemia. Exploring the optimal quantity of oyster consumption for humans holds the potential for effectively combating anemia.

## 5. Conclusions

The results demonstrate that oral oyster supplementation is an effective way of correcting anemia in experimental anemic rats.

The high zinc content in oysters is pivotal in their effectiveness for correcting anemia in experimental rats.

The effectiveness of zinc supplementation is influenced by the method of administration, the zinc dosage, and the specific zinc form used.

Oysters not only possess significantly elevated zinc levels but also contain a substantial amount of iron. Oral oyster supplementation appears to be a promising strategy for addressing anemia in rats.

## Figures and Tables

**Table 1:** Various zinc supplementation methods and their source for phenylhydrazine-induced anemic rats.

Group	Zn Supplemented	Source
(a) Injection of saline	0	Market diet only
(b) Injection of ZnSO_4_	0.95 mg Zn/rat	Market diet + 1 injection of 4.18 mg ZnSO_4_·7H_2_O/rat
(c) Fed with ZnSO_4_	2.70 mg Zn/day/rat	Market diet + 11.88 mg ZnSO_4_·7H_2_O/day/rat
(d) Fed with oyster	2.70 mg Zn/day/rat	Market diet + 30 g oyster/day/rat

The average body weight of the rats was 339 ± 17 g (mean ± SD).

## Data Availability

The data are available from the corresponding author.

## Supplementary Materials

The following supporting information can be downloaded at: https://www.mdpi.com/article/10.3390/nu15214675/s1, Table S1. Hematology of the normal rats, phenylhydrazine (PHZ induced rats and anemic rats with different zinc supplementation. Table S2. Hematology of the oral ZnSO_4_ 7H_2_O and oyster supplemented 5/6 nephrectomized anemic rats. Table S3. Plasma zinc level in blood of the oral ZnSO_4_, and oyster supplemented 5/6 nephrectomized anemic rats. Table S4. Hematology of the oral ZnSO_4_, oyster, or hard clam supplemented 5/6 nephrectomized anemic rats.
