# Supplementary material for: Oral Zinc-Rich Oyster Supplementation Corrects Anemia in Rats
Source: Nutrients. 2023 Nov 4;15(21):4675. doi: 10.3390/nu15214675 (PMC10650441; doi:10.3390/nu15214675)
Supplement: Supplementary file 1 [file nutrients-15-04675-s001.zip › Table S1.pdf]

Table S1. Hematology of the normal rats, phenylhydrazine (PHZ)-induced rats and anemic rats with different zinc supplementation

Rat group	RBC ($10^6/mm^3$)	HCT (%)	Hb (g/dl)	MCV (μm^3)	MCH (pg/cell)	MCHC (g/dl)
1. d0						
Normal rats						
(n=6)	7.00 ± 0.18	46.6 ± 1.1	15.2 ± 0.5	67 ± 2	22 ± 1	33 ± 1
2. d2						
Saline injected rats						
(n=6)	6.99 ± 0.27	40.5 ± 1.7	14.0 ± 0.5	58 ± 2	20 ± 1	35 ± 1
PHZ-induced anemic rats						
(n=24)	5.01 ± 0.79	37.2 ± 4.4	14.9 ± 2.2	75 ± 11	30 ± 6	40 ± 4
3. d6						
4 days after zinc supplementation						
(a) Saline-injected						
(n=6)	4.46 ± 0.30	33.3 ± 1.4	11.9 ± 0.7	75 ± 4	27 ± 1	36 ± 1
(b) $ZnSO_4$-injected						
(n=6)	5.65 ± 0.56**	37.8 ± 0.6***	13.2 ± 0.1**	68 ± 6*	24 ± 2*	35 ± 4
(c) Fed with $ZnSO_4 \cdot 7H_2O$						
(n=6)	4.51 ± 0.42	34.0 ± 1.9	11.9 ± 0.7	76 ± 8	26 ± 3	35 ± 1
(d) Fed with oyster						
(n=6)	5.81 ± 0.55***	38.9 ± 1.4***	13.9 ± 0.6***	67 ± 6*	24 ± 2**	36 ± 1

RBC total red blood cell count, HCT hematocrit, Hb hemoglobin, MCV mean corpuscular volume concentration (HCT/RBC × 10), MCH mean corpuscular hemoglobin (Hb/RBC × 100), MCHC mean corpuscular hemoglobin concentration (Hb/HCT × 100)

The data are expressed as the mean ± SD

(* $p < 0.05$, ** $p < 0.01$, *** $p < 0.001$ Compared with the saline-injected group)
