# Supplementary material for: Oral Zinc-Rich Oyster Supplementation Corrects Anemia in Rats
Source: Nutrients. 2023 Nov 4;15(21):4675. doi: 10.3390/nu15214675 (PMC10650441; doi:10.3390/nu15214675)
Supplement: Supplementary file 1 [file nutrients-15-04675-s001.zip › Table S2.pdf]

Table S2. Hematology of the oral $ZnSO_4 \cdot 7H_2O$ and oyster-supplemented 5/6 nephrectomized anemic rats

Rat group	RBC ($10^6/mm^3$)	HCT (%)	Hb (g/dl)	MCV (μm^3)	MCH (pg/cell)	MCHC (g/dl)
1. d0						
Normal rats (n=6)	7.13 ± 0.31	40.9 ± 2.4	14.8± 0.8	57± 3	21± 1	26± 1
2. d25						
Normal rats (n=6)	6.81± 0.56	45.8 ± 1.3	17.7± 0.4	68± 4	26± 2	39± 1
5/6 Nephrectomized rats (n=30)	5.75 ± 0.40	37.8 ± 3.2	16.6 ± 1.1	67± 6	29± 4	44± 4
3. A group						
Normal rats (+0 mg Zn supplementation)						
0 weeks (n=6)	6.81 ± 0.56	45.8 ± 1.3	17.7 ± 0.4	68 ± 4	26 ± 2	39 ± 1
2 " (n=6)	7.28 ± 0.47	46.8 ± 1.8	17.6 ± 0.6	64 ± 3	24 ± 1	38 ± 2
4 " (n=6)	7.38 ± 0.55	46.8 ± 2.2	17.3 ± 0.3	64 ± 3	24 ± 1	37 ± 1
6 " (n=6)	7.26 ± 0.61	45.9 ± 3.1	17.4 ± 0.8	63 ± 3	24 ± 2	38 ± 2
8 " (n=6)	7.41 ± 0.74	45.8 ± 3.0	18.2 ± 0.8	62 ± 4	25 ± 2	40 ± 2
4. B-a group						
5/6 Nephrectomized rats (+0 mg Zn supplementation)						
0 weeks (n=6)	5.75 ± 0.38	38.0 ± 3.7	13.6 ± 1.2	66 ± 2	24 ± 1	36 ± 1
2 " (n=6)	5.87 ± 0.45	37.4 ± 2.4	13.7 ± 0.8	64 ± 2	23 ± 1	37 ± 1
4 " (n=6)	5.99 ± 0.26	37.6 ± 0.8	13.7 ± 0.4	63 ± 2	23 ± 1	36 ± 0
6 " (n=6)	5.67 ± 0.49	36.0 ± 2.3	13.2 ± 0.8	63 ± 2	23 ± 1	37 ± 1
8 " (n=6)	5.00 ± 0.77	31.9 ± 4.1	11.5 ± 1.4	64 ± 3	23 ± 1	36 ± 1
5. B-b group						
5/6 Nephrectomized rats (+1.35 mg Zn (5.94 mg $ZnSO_4 \cdot 7H_2O$)/day/rat supplementation)						
0 weeks (n=6)	5.93 ± 0.66	37.9 ± 1.5	16.5 ± 0.8	65 ± 7	28 ± 2	43 ± 2
2 " (n=6)	5.86 ± 0.22	38.9 ± 1.0	16.0 ± 0.2	66 ± 2	27 ± 1	41 ± 2
4 " (n=6)	5.80 ± 0.52	38.0 ± 1.8	15.3± 0.7	66 ± 4	27 ± 2	40 ± 1
6 " (n=6)	5.60 ± 0.59	37.9 ± 3.1	15.7 ± 0.6	68± 2	28 ± 2	42 ± 3
8 " (n=6)	5.70 ± 1.12	37.4± 6.0	16.2 ± 1.5	66 ± 3	29 ± 3	44 ± 3
6. B-c group						
5/6 Nephrectomized rats (+2.70 mg Zn (11.88 mg $ZnSO_4 \cdot 7H_2O$)/day/rat supplementation)						
0 weeks (n=6)	5.85 ± 0.29	38.5 ± 4.3	16.7 ± 0.8	66 ± 5	29 ± 2	44 ± 4
2 " (n=6)	6.36 ± 0.55*	41.6 ± 3.4*	16.4 ± 1.0	66 ± 2	26 ± 1	40 ± 2
4 " (n=6)	6.05 ± 0.78	40.0 ± 5.1	15.7 ± 0.7	66 ± 2	26 ± 3	40 ± 5
6 " (n=6)	5.98 ± 0.78	39.7 ± 4.5	16.6 ± 1.0	67 ± 2	28 ± 2	42 ± 2
8 " (n=6)	6.18 ± 0.76	40.1± 3.9	16.3 ± 1.2	65 ± 2	27 ± 2	41 ± 2

7. B-d group						
5/6 Nephrectomized rats (+1.35 mg Zn (15 g oyster)/day/rat supplementation)						
0 weeks (n=6)	5.52 ± 0.48	37.3 ± 0.9	16.3 ± 0.7	67 ± 2	30 ± 1	44 ± 2
2 " (n=6)	5.77 ± 0.69	39.2 ± 3.0	15.7 ± 0.4	68 ± 1	27 ± 2	40 ± 2
4 " (n=6)	6.27 ± 0.79*	41.6 ± 3.6	16.2 ± 0.9	67 ± 2	26 ± 2	39 ± 1
6 " (n=6)	6.09 ± 0.41*	40.5 ± 2.3	16.9 ± 0.5	67 ± 1	28 ± 2	42 ± 2
8 " (n=6)	5.91 ± 0.96	38.9 ± 3.8	15.8 ± 1.3	66 ± 3	27 ± 2	41 ± 1
8. B-e group						
5/6 Nephrectomized rats (+2.70 mg Zn (30 g oyster)/day/rat supplementation)						
0 weeks (n=6)	5.69 ± 0.32	37.3 ± 1.1	16.8 ± 0.9	66 ± 2	30 ± 2	45 ± 3
2 " (n=6)	6.21 ± 0.31**	41.8 ± 1.9***	16.5 ± 0.6	68 ± 1	27 ± 1	40 ± 2
4 " (n=6)	6.16 ± 0.40*	41.2 ± 2.9*	16.4 ± 0.7	67 ± 2	27 ± 1	40 ± 2
6 " (n=6)	6.18 ± 0.51*	40.6 ± 1.8**	17.0 ± 0.7	66 ± 3	28 ± 1	42 ± 1
8 " (n=6)	6.63 ± 0.49**	42.0 ± 2.7**	16.7 ± 0.6	64 ± 4	25 ± 1	40 ± 2

RBC total red blood cell count, HCT hematocrit, Hb hemoglobin, MCV mean corpuscular volume (HCT/RBC × 10), MCH mean corpuscular hemoglobin (Hb/RBC × 100), MCHC mean corpuscular hemoglobin concentration (Hb/HCT×100)

The data are expressed as the mean ± SD

(* $p < 0.05$, ** $p < 0.01$, *** $p < 0.001$ Compared with the 0-week group).

This table represents two independent experiments.
