# Supplementary material for: Oral Zinc-Rich Oyster Supplementation Corrects Anemia in Rats
Source: Nutrients. 2023 Nov 4;15(21):4675. doi: 10.3390/nu15214675 (PMC10650441; doi:10.3390/nu15214675)
Supplement: Supplementary file 1 [file nutrients-15-04675-s001.zip › Table S3.pdf]

Table S3. Plasma zinc level in blood of the oral $ZnSO_4 \cdot 7H_2O$, and oyster-supplemented 5/6 nephrectomized anemic rats

Rat group	Plasma Zn level in blood (μg/mL plasma)	*p*
1. A group		
Normal rats (+0 mg Zn supplementation)		
0 weeks (n=6)	1.70 ± 0.17	
2 " (n=6)	1.59 ± 0.10	0.1680
4 " (n=6)	1.88 ± 0.16	0.0522
6 " (n=6)	1.93 ± 0.14	0.0464*
8 " (n=6)	1.72 ± 0.19	0.8694
2. B-b group		
5/6 Nephrectomized rats (+1.35 mg Zn (5.94 mg $ZnSO_4 \cdot 7H_2O$)/day/rat supplementation)		
0 weeks (n=6)	1.53 ± 0.16	
2 " (n=6)	1.51 ± 0.09	0.7316
4 " (n=6)	1.80 ± 0.13	0.0147*
6 " (n=6)	1.59 ± 0.12	0.3526
8 " (n=6)	1.52 ± 0.11	0.9128
2. B-c group		
5/6 Nephrectomized rats (+2.70 mg Zn (11.88 mg $ZnSO_4 \cdot 7H_2O$)/day/rat supplementation)		
0 weeks (n=6)	1.47 ± 0.05	
2 " (n=6)	1.36 ± 0.23	0.3879
4 " (n=6)	1.66 ± 0.06	0.0132*
6 " (n=6)	1.57 ± 0.09	0.1147
8 " (n=6)	1.52 ± 0.11	0.5543
4. B-d group		
5/6 Nephrectomized rats (+1.35 mg Zn (15 g oyster)/day/rat supplementation)		
0 weeks (n=6)	1.54 ± 0.07	
2 " (n=6)	1.62 ± 0.26	0.5267
4 " (n=6)	1.71 ± 0.13	0.0815
6 " (n=6)	1.72 ± 0.11	0.0754
8 " (n=6)	1.63 ± 0.13	0.3041
5. B-e group		
5/6 Nephrectomized rats (+2.70 mg Zn (30 g oyster)/day/rat supplementation)		
0 weeks (n=6)	1.53 ± 0.12	
2 " (n=6)	1.61 ± 0.11	0.2353
4 " (n=6)	1.66 ± 0.12	0.0565
6 " (n=6)	1.67 ± 0.15	0.0360*
8 " (n=6)	1.57 ± 0.26	0.7981

The data are expressed as the mean±SD (*p*, compared with the 0 week group, *$p \leq 0.05$)
