# Supplementary material for: Oral Zinc-Rich Oyster Supplementation Corrects Anemia in Rats
Source: Nutrients. 2023 Nov 4;15(21):4675. doi: 10.3390/nu15214675 (PMC10650441; doi:10.3390/nu15214675)
Supplement: Supplementary file 1 [file nutrients-15-04675-s001.zip › Table S4.pdf]

Table S4. Hematology of the oral $ZnSO_4 \cdot 7H_2O$, oyster, or hard clam-supplemented 5/6 nephrectomized anemic rats

Rat group	RBC ($10^6/mm^3$)	HCT (%)	Hb (g/dl)	MCV (μm^3)	MCH (pg/cell)	MCHC (g/dl)
1. d0						
Normal rats (n=6)	7.91 ± 0.19	48.8 ± 0.4	15.9 ± 0.6	62 ± 2	20 ± 1	33 ± 1
2. d25						
Normal rats (n=6)	7.78 ± 0.38	55.1 ± 2.7	16.8 ± 0.5	71 ± 4	22 ± 1	31 ± 2
5/6 Nephrectomized rats (n=18)	6.27 ± 0.29	42.3 ± 1.8	15.8 ± 0.6	68 ± 3	25 ± 1	37 ± 2
3. A group						
Normal rats (+0 mg Zn supplementation)						
0 weeks (n=6)	7.78± 0.38	55.1 ± 2.7	16.8 ± 0.5	71 ± 4	22 ± 1	31 ± 2
2 " (n=6)	7.65 ± 0.40	48.7 ± 1.7	16.5 ± 0.4	64 ± 3	22 ± 1	34 ± 1
4 " (n=6)	7.79 ± 0.23	47.7 ± 2.6	16.7 ± 0.3	61 ± 2	21 ± 1	35 ± 1
6 " (n=6)	7.95 ± 0.32	51.4 ± 1.3	16.6 ± 0.5	65 ± 3	21 ± 1	32 ± 1
8 " (n=6)	7.69 ± 0.30	49.1 ± 1.0	16.5 ± 0.5	64 ± 2	21 ± 1	34 ± 1
4. B-a group						
5/6 Nephrectomized rats (+2.70 mg Zn (5,94 mg $ZnSO_4 \cdot 7H_2O$)/day/rat supplementation)						
0 weeks (n=6)	6.26 ± 0.34	42.9 ± 1.1	15.6 ± 0.5	69 ± 3	25 ± 1	36 ± 1
2 " (n=6)	6.17 ± 0.15	41.2 ± 1.5	15.1 ± 0.9	67 ± 2	24 ± 1	37 ± 2
4 " (n=6)	6.15 ± 0.25	42.0 ± 1.6	15.8 ± 0.7	68 ± 2	26 ± 1	38 ± 1
6 " (n=6)	6.43 ± 0.34	43.4 ± 1.9	15.9 ± 0.5	68 ± 2	25 ± 1	37 ± 2
8 " (n=6)	5.81 ± 0.28	39.5 ± 1.6	14.1 ± 0.8	68 ± 3	24 ± 1	36 ± 1
5. B-b group						
5/6 Nephrectomized rats (+2.70 mg Zn (30 g oyster)/day/rat supplementation)						
0 weeks (n=6)	6.22 ± 0.24	41.6 ± 1.1	15.9 ± 0.7	67 ± 2	26 ± 1	38 ± 2
2 " (n=6)	6.76 ± 0.24**	41.4 ± 1.2	15.2 ± 0.7	61 ± 3	22 ± 1	37 ± 2
4 " (n=6)	6.87 ± 0.17**	42.2 ± 2.6	15.7 ± 0.7	61 ± 3	23 ± 1	37 ± 1
6 " (n=6)	6.92 ± 0.38**	42.5 ± 2.0	15.9 ± 1.0	61 ± 3	23 ± 1	37 ± 1
8 " (n=6)	6.85 ± 1.12**	41.3 ± 1.7	15.4 ± 0.9	60 ± 3	23 ± 1	37 ± 1
6. B-c group						
5/6 Nephrectomized rats (+0.42 mg Zn (30 g hard clam)/day/rat supplementation)						
0 weeks (n=6)	6.32 ± 0.25	42.4 ± 2.5	15.9 ± 0.2	67 ± 4	25 ± 1	38 ± 2
2 " (n=6)	5.98 ± 0.49	39.7 ± 0.9	15.3 ± 0.7	66 ± 3	26 ± 1	38 ± 1
4 " (n=6)	6.08 ± 0.32	40.2 ± 1.1	15.5 ± 0.8	66 ± 2	25 ± 2	38 ± 1
6 " (n=6)	6.12 ± 0.27	41.4 ± 2.6	15.5 ± 1.1	68 ± 2	25 ± 1	37 ± 2
8 " (n=6)	5.72 ± 0.54	38.8 ± 2.4	14.2 ± 1.4	68 ± 3	25 ± 1	37 ± 2

RBC total red blood cell count, HCT hematocrit, Hb hemoglobin, MCV mean corpuscular volume (HCT/RBC

× 10), MCH mean corpuscular hemoglobin (Hb/RBC × 100), MCHC mean corpuscular hemoglobin concentration (Hb/HCT×100)

The data are expressed as the mean ± SD

(* $p < 0.05$, ** $p < 0.01$, *** $p < 0.001$ Compared with the 0-week group)
